# How can we identify subglottic stenosis in patients with suspected obstructive disease?

**DOI:** 10.1007/s00405-023-08141-3

**Published:** 2023-08-04

**Authors:** Eleftherios Ntouniadakis, Josefin Sundh, Jeanette Söderqvist, Mathias von Beckerath

**Affiliations:** 1 Department of Ear Nose and Throat, Faculty of Medicine and Health, Örebro University Hospital, Södra Grev Rosengatan, 701 85 Örebro, Sweden; 2 Department of Respiratory Medicine, Faculty of Medicine and Health, Örebro University, 70182 Örebro, Sweden; 3 Department of Clinical Physiology, Faculty of Medicine and Health, Örebro University, 70182 Örebro, Sweden; 4 Department of Clinical Sciences, Intervention and Technology, Karolinska Institutet, Karolinska University Hospital, Stockholm, Sweden

**Keywords:** Subglottic stenosis, Expiratory Disproportion Index, Dyspnea Index, Functional assessment, Asthma, COPD

## Abstract

**Purpose:**

Subglottic stenosis, a rare condition of the upper airway, is frequently misdiagnosed as obstructive lung disease. The aim of this study was to investigate whether subglottic stenosis could be identified and distinguished from asthma and chronic obstructive pulmonary disease (COPD) using spirometry or the dyspnea index (DI).

**Methods:**

The study population included 43 patients with asthma, 31 patients with COPD and 50 patients with subglottic stenosis planned to undergo endoscopic intervention. All patients completed the DI and underwent dynamic spirometry registering both inspiratory and expiratory volumes and flows, including the expiratory disproportion index (EDI), the ratio of forced expiratory volume in 1 s to peak expiratory flow. One-way analysis of variance assessed the discrepancy of the variables among the study groups, and receiver operating curve (ROC) analysis determined the measurement with the best discriminatory power providing a cutoff value, maximizing both sensitivity and specificity.

**Results:**

The only statistically significant variables differing between all three groups were the EDI and the DI. The EDI showed an excellent area under the ROC curve (0.99, *p* < 0.001) with a cutoff value of 0.39 (98% sensitivity, 96% specificity), followed by DI (0.87, *p* < 0.001) with a cutoff score of > 25 (83% sensitivity and 78% specificity).

**Conclusion:**

In patients with dyspnea of unknown cause, an increase in EDI should arouse a suspicion of extrathoracic airway obstruction, advocating for further evaluation with laryngotracheoscopy.

**Supplementary Information:**

The online version contains supplementary material available at 10.1007/s00405-023-08141-3.

## Introduction

Subglottic stenosis (SGS) is a gradual narrowing of the airway caused by chronic inflammation of the tracheal mucosa below the vocal folds, presenting with relatively common symptoms during its early process, such as dyspnea at exertion, wheezing, chronic cough or dysphonia, and stridor in cases of severe obstruction [1]. With an incidence of up to 1:200,000 regardless cause [1–3], it is frequently misinterpreted as asthma or chronic obstructive pulmonary disease (COPD), resulting in a reported mean time to definite diagnosis of more than 2 years from the onset of symptoms, partly due to the concealed measurements in dynamic spirometry [4–6].

Flexible laryngotracheoscopy, although an invasive procedure, is considered the gold-standard examination and is imperative to set the diagnosis of SGS [4, 5, 7–9]. Visual changes in the inspiratory and expiratory loop in spirometry is the most prevailing sign of upper airway obstruction attainable in the clinical setting [10–12]. The expiratory disproportion index (EDI), the ratio of forced expiratory volume in 1 s (FEV_1_) to the peak expiratory flow (PEF), when found greater than 0.50 could indicate extrathoracic airway obstruction [13–15]. Although it was introduced at the early 1970s [13, 15], and despite the solid findings from Nouraei et al. [14], it is indeed not acknowledged in the differential diagnostics of dyspnea of unknown cause. Some studies discuss the usefulness of spirometry by measuring EDI, PEF, peak inspiratory flow (PIF) or total peak flow (TPF), alone or combined with a subjective assessment with the dyspnea index (DI), in the evaluation of treatment response or disease progression of SGS [16–20]. The DI is a 10-item, five-point Likert questionnaire with a total sum ranging from 0 to 40, uniquely developed for and used in assessing upper airway dyspnea, where a higher score represents more severe symptoms (Additional files 1 and 2) [21, 22].

In summary, the optimal initial investigations to identify patients in need of laryngotracheoscopy presenting with dyspnea refractory to conventional treatment are still not established. Thus, the aim of this study was to identify patterns in spirometry or DI scoring that could distinguish patients with SGS from patients with asthma and COPD, contributing to the correct detection of the disorder without a diagnostic delay.

## Methods

Adult patients with SGS who were scheduled to undergo endoscopic treatment at the Ear Nose and Throat Department at Örebro University Hospital were consecutively included from September 2016 to December 2020. Exclusion criteria were a stricture caused by an external compression of the trachea, malignant tumors, or those with multilevel stenosis further engaging the glottic or supraglottic part of the airway. In the same manner, patients with asthma and COPD referred to the Department of Respiratory Medicine at Örebro University Hospital were consecutively included between 2020 and 2021.

All study subjects completed the Swedish version of the DI and underwent spirometry prior to treatment. Spirometry was performed by certified health care professionals from the Department of Clinical Physiology, either at our hospital or at referral hospitals. The following variables were registered: DI score, FEV_1_, PEF, PIF, TPF, forced inspiratory volume in 1 s (FIV_1_), EDI, Forced Vital Capacity (FVC), FEV_1_/FVC ratio and percent of the predicted FEV_1_ (FEV_1_%) and FVC (FVC%) value according to the Global Lung Function Initiative. FVC was replaced with vital capacity when FVC values were missing. The Shapiro‒Wilk test was performed to investigate the normality of the baseline characteristics and all variables. Normally distributed continuous variables are presented using the means and standard deviation (SD), nonnormally distributed variables with the medians and interquartile range (IQR), and categorical variables as the numbers and percentages.

One-way analysis of variance (ANOVA) including Bonferroni post hoc analysis was used to evaluate the discrepancy of the variables among the three study groups. The diagnostic value of each variable was investigated with receiver operating curve (ROC) analysis by comparing the area under the ROC curve (AUC), which represents the test’s discriminatory power. A ROC curve comprises all different values of each variable according to sensitivity (placed on the *y*-axis) and 1-specificity (placed on the *x*-axis). The AUC is categorized as excellent (0.90 < AUC < 1.00), good (0.8 < AUC < 0.89), fair (0.70 < AUC < 0.79), poor (0.60 < AUC < 0.69) and failure (0.50 < AUC < 0.59). We further sought to extract a cutoff value, maximizing both sensitivity and specificity for the variables showing at least a good AUC. This value providing balanced sensitivity and specificity is defined as the point on the apex of the ROC curve, being the highest point of the vertical axis and further to the left on the horizontal axis [23].

Due to the large number of assessed variables, the Bonferroni equation of *α/n* = 0.05 was used to calculate the *p* value. As the number of assessed variables was eleven, a *p* value of 0.005 was considered statistically significant.

IBM^®^ SPSS^®^ Statistics software, version 27 (Armonk, NY, USA; IBM Corp.) was used for the statistical analysis. This human study was performed in accordance with the Declaration of Helsinki Guidelines and was approved by the Ethics Review Board in Uppsala, diary number 2016/193. An amendment to include patients with asthma and COPD was approved by the Swedish Ethical Review Authority, diary number 2020-05509. All adult participants provided written informed consent to participate.

## Results

### Patient characteristics

In total, 50 patients with SGS, 43 with COPD and 31 with asthma were included. The demographic data of the study population at baseline are listed in Table 1. We also present information regarding smoking history, diabetes, and presence of cardiovascular comorbidities, defined as ischemic heart disease, heart failure, arrhythmia, or cerebrovascular condition. Detailed lesion characteristics in patients with stenosis are presented in Table 2. Spirometry measurements in each cohort are shown in Table 3.

**Table 1:** Demographic data of the study group

Diagnosis	SGS	Asthma	COPD
Sex *n* (%)			
Male	4 (8%)	16 (51.6%)	11 (25.6%)
Female	46 (92%)	15 (48.4%)	32 (74.4%)
Age mean (SD)	56.3 (13.7)	54.7 (14.3)	70.6 (8.6)
Smoking history			
Current smoker	3 (6%)	2 (6.7%)	5 (11.4%)
Never smoker	43 (86%)	17 (56.7%)	3 (6.8%)
Former smoker	4 (8%)	11 (36.7%)	36 (81.8%)
Diabetes			
Positive	6 (12%)	1 (3.3%)	5 (11.4%)
Negative	44 (88%)	29 (96.7%)	39 (88.6%)
Cardiovascular comorbidities			
Positive	0	4 (13.3%)	11 (25%)
Negative	50 (100%)	26 (86.7%)	33 (75%)
BMI			
Mean (SD)	29.0 (6.7)	30.2 (6.0)	24.8 (6.0)
< 20	3 (6%)	0	9 (20.5%)
20–24.9	14 (28%)	5 (16.7%)	15 (34.1%)
25–29.9	15 (30%)	8 (26.7%)	11 (25%)
> 30	18 (36%)	17 (56.7%)	9 (20.5%)

*SD* standard deviation, *BMI* body mass index

**Table 2:** Lesion characteristics in patients with subglottic stenosis

Etiology	
Idiopathic	39 (78%)
GPA	1 (2%)
Positive autoimmune serology—ANCA-negative	6 (12%)
Rheumatoid arthritis	3 (5%)
Prolonged intubation	1 (2%)
Intubation history within 2 years prior to diagnosis setting	
Positive	13 (26%)
Negative	37 (74%)
Cotton–Myer grade	
I	7 (14%)
II	29 (58%)
III	14 (28%)

*GPA* granulomatosis with polyangiitis, *ANCA* antineutrophil cytoplasmic antibodies

**Table 3:** Pulmonary function data in different patient groups

Mean (SD)	SGS	Asthma	COPD
DI	30.6 (5.8)	16.2 (8.1)	20.7 (8.1)
FEV_1_ (L)	2.3 (0.6)	2.7 (1.0)	1.1 (0.5)
FEV_1_%	74.9 (16.8)	87.3 (22.3)	42.1 (19.4)
FIV_1_ (L)	2.2 (0.7)	3.5 (1.3)	2.0 (0.7)
PEF (L/s)	4.0 (1.4)	8.5 (2.9)	3.6 (2.1)^a^
PIF (L/s)	2.7 (0.9)	6.2 (2.2)	3.4 (1.4)
TPF (L/s)	6.6 (2.2)	14.7 (4.9)	7.2 (2.7)
FVC (L)	3.4 (0.8)	3.8 (1.3)	2.4 (0.8)
FVC %	85.6 (13.6)	96.5 (24.4)	72.0 (24.2)
FEV_1_/FVC	0.73 (0.12)^a^	0.72 (0.06)	0.45 (0.12)
EDI	0.59 (0.30)^a^	0.32 (0.06)^a^	0.28 (0.07)

*L* liter, *L/s* liter/second

^a^nonnormally distributed data are presented with median (interquartile range)

### Differences in spirometry and DI values in stenosis vs. nonstenosis cohorts

A one-way ANOVA indicated that there was a statistically significant difference in the mean value of all study variables between at least two cohorts; however, the only mean values that differed significantly between both the nonstenosis groups and the stenosis cohort were DI and EDI, as shown in Table 4. FEV_1_/FVC was significantly reduced in patients with COPD compared with patients with asthma or stenosis. In contrast, PIF, PEF and TPF were all significantly lower in stenosis than in asthma but did not differ from the COPD group.

**Table 4:** One-way ANOVA results comparing the difference between variables in all study cohorts

Variable	*F* value (between groups *df*, within groups *df*)	*p* value	Comparison between groups		Mean difference	*p* value	95% CI
DI	*F* 42.224 (2,120)	< 0.001	SGS	Asthma	14.4	< 0.001	10.4 to 18.5
				COPD	9.9	< 0.001	6.2 to 13.6
FEV_1_ (L)	*F* 62.205 (2,121)	< 0.001	SGS	Asthma	− 0.42	< 0.26	− 0.8 to − 0.04
				COPD	1.3	< 0.001	0.9 to 1.6
FEV_1_%	*F* 57.709 (2,121)	< 0.001	SGS	Asthma	− 6.5	0.6	− 18.7 to 5.7
				COPD	35.4	< 0.001	24.3 to 46.5
FIV_1_ (L)	*F* 28.466 (2,121)	< 0.001	SGS	Asthma	− 1.2	< 0.001	− 1.7 to − 0.8
				COPD	0.2	0.70	− 0.2 to 0.7
PEF (L/s)	*F* 66.394 (2,121)	< 0.001	SGS	Asthma	− 4.5	< 0.001	− 5.6 to − 3.5
				COPD	0.1	1.0	− 0.9 to 1.1
PIF (L/s)	*F* 55.731 (2,120)	< 0.001	SGS	Asthma	− 3.5	< 0.001	− 4.3 to − 2.7
				COPD	− 0.7	.076	− 1.5 to 0.1
TPF (L/s)	*F* 67.728 (2,120)	< 0.001	SGS	Asthma	− 8.1	< 0.001	− 9.9 to − 6.3
				COPD	− 0.6	1	− 2.3 to 1.0
FVC (L)	*F* 23.004 (2,118)	< 0.001	SGS	Asthma	− 0.4	0.15	− 1 to 0.1
				COPD	1.0	< 0.001	0.5 to 1.5
FVC %	*F* 12.806 (2,118)	< 0.001	SGS	Asthma	− 11.0	0.074	− 22.6 to 0.7
				COPD	13.5	= 0.008	2.9 to 24.1
FEV_1_/FVC	*F* 89.656 (2,118)	< 0.001	SGS	Asthma	0	1.0	− 0.07 to 0.04
				COPD	0.26	< 0.001	0.20 to 0.31
EDI	*F* 119.265 (2,121)	< 0.001	SGS	Asthma	0.31	< 0.001	0.25 to 0.38
				COPD	0.36	< 0.001	0.30 to 0.42

*F* variation between sample means/variation within the samples, *df* degrees of freedom, *CI* confidence intervals, *L* liter, *L/s* liter/second

### Assessing the diagnostic ability of spirometry measures and DI

**Fig. 1:**
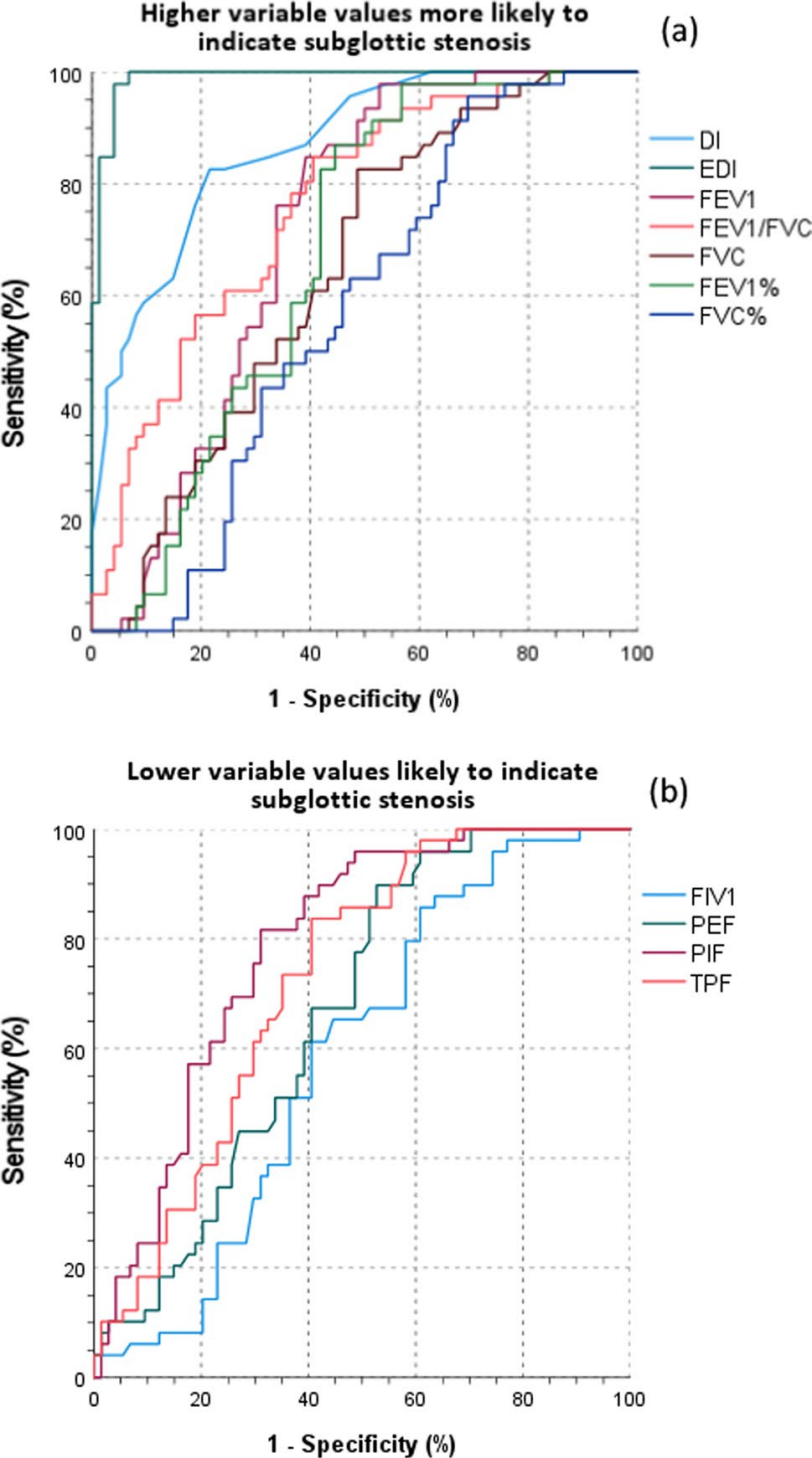
Visual representations of ROC analysis for all study variables. Variables where higher values are more likely to indicate stenosis (**a**) and variables where lower values are more likely to indicate stenosis (**b**)

The ROC analysis revealed an excellent AUC for EDI (AUC = 0.99), with an optimal cutoff value of greater than 0.39, showing 98% sensitivity and 96% specificity. The DI showed a good AUC = 0.87 and a cutoff score > 25, yet with 83% sensitivity and 78% specificity. ROC analysis with AUC and a visual representation of the ROC curve for each study variable are fully presented in Table 5 and Fig. 1, respectively.

**Table 5:** ROC analysis for all study variables, including AUC and cutoff values maximizing sensitivity and specificity

	AUC	95% CI	*p* value	Cutoff value	Sensitivity (%)	Specificity (%)
Higher variable values likely to indicate subglottic stenosis						
EDI	0.99	0.98–1.00	< 0.001	> 0.39	98	96
DI	0.87	0.81–0.93	< 0.001	> 25	83	78
FEV_1_/FVC	0.76	0.68–0.85	< 0.001	> 0.65	80	61
FEV_1_	0.72	0.63–0.81	< 0.001	> 1.9 L	76	66
FEV_1_%	0.68	0.58–0.77	< 0.001	> 62.9%	83	58
FVC	0.65	0.55–0.74	= 0.007	> 3.0 L	61	59
FVC %	0.57	0.47–0.67	0.191	> 83.4%	61	54
Lower variable values likely to indicate subglottic stenosis						
PIF	0.79	0.71–0.87	< 0.001	< 3.23 L/s	76	70
TPF	0.73	0.64–0.81	< 0.001	< 7.83 L/s	74	65
PEF	0.67	0.57–0.76	< 0.001	< 4.43 L/s	67	59
FIV_1_	0.59	0.49–0.69	= 0.084	< 2.32 L	65	55

*ROC* receiver operating curve, *AUC* area under the curve, *CI* confidence intervals 95%, *L* liter, *L/s* liter/second

## Discussion

The primary finding of this study suggests that an EDI with a value > 0.39 is the optimal spirometry measurement, showing high sensitivity and specificity for differentiating stenosis from the nonstenosis cohort. Secondary findings are that a DI score > 25 may also contribute to the differentiation of the stenosis against nonstenosis groups and that a reduced PIF or TPF could be used to distinguish stenosis from asthma in patients with a normal FEV_1_/FVC ratio.

Shortness of breath not responding to conventional treatment is challenging for every physician. SGS is commonly misinterpreted as “difficult-to-treat asthma” or other causes of lower airway obstruction, such as COPD and bronchitis, resulting in a diagnostic delay because of the rarity of the condition and the overlapping clinical presentation of upper airway obstruction with other causes of dyspnea [4, 24]. It would indeed be favorable to detect a functional deterioration of the upper airway with a nontraumatic examination, such as spirometry, particularly when the incidence of stenosis is expected to rise for the following years after the COVID-19 pandemic [25]. Hence, the flow-volume loops and the inspiratory part of the test, in particular inspiratory flow rates, have traditionally been used to identify this condition. Undesirably, inspiratory maneuvers are not included in standard dynamic spirometry and are therefore less feasible for diagnostics outside physiology departments.

Other reported potential measurements proposed for diagnosing extrathoracic airway obstruction are the ratio of maximal expiratory flow at 50% of FVC to maximal inspiratory flow at 50% of FVC less than 0.30 or more than 1 [10], a PIF less than 100 L/min [15] and the ratio of FEV_1_ to forced expiratory volume in 0.5 s greater than 1.5 [15]. Nevertheless, these are sophisticated values that are not routinely extracted from the test; some require an inspiratory maneuver and are therefore difficult to implement in daily praxis [26].

In COPD, both PEF and PIF may also be reduced, depending on the increased airway resistance that reflects parenchymal inflammation [27–30]. However, in SGS, the total resistance of the airway is increased, resulting in a reduced initial airflow, which is clearly visualized at the flow-volume loops with a flattened expiratory curve, and a reduction of the PEF [12, 31]. This is consistent with our findings that PEF, PIF, TPF, and FIV_1_ were significantly lower in stenosis group and COPD than in asthma, with nonsignificant differences between stenosis and COPD.

FEV_1_ in asthmatic patients is often normal in stable situations, is reduced in COPD in moderate to very severe disease (i.e., COPD stages 2–4) but remains normal in stage 1, corresponding to mild severity. This rationale is also fully in agreement with our findings, where FEV_1_% was nonsignificantly decreased in stenosis and clearly significantly decreased in COPD compared with the normal mean FEV_1_% in the asthma group. FEV_1_ should be normal in upper airway obstruction since it is determined by the status of the small intrathoracic airways, which remain unaffected even in cases of substantial reduction of the tracheal lumen [12].

The unproportioned change of a substantially diminished PEF in relation to a relatively unchanged FEV_1_, provided no parenchymal inflammation, comprises the theoretical background of the EDI: the ratio of FEV_1_ (measured in liters, L) to PEF (L/s). It was first described by Empey et al. [13], and was later supported by the solid results from Nouraei et al. [14]. These groundbreaking works are further consolidated by our data, extending the evidence that EDI could differentiate particularly SGS from both asthma and COPD, the two diagnoses implicated in the diagnostic delay of this condition. Together with other studies discussing EDI’s role in monitoring the treatment effects of patients with subglottic stenosis, our study clearly spotlights the main benefit of extracting the EDI routinely from conventional spirometry [14, 19, 20, 32].

Since it can be easily calculated from data recorded in dynamic spirometry, which is performed at all primary health care centers, we believe it offers a convenient and feasible way to screen patients with suspected SGS, where further assessment with laryngotracheoscopy is needed. In addition, EDI seems to be superior to other spirometry measurements requiring an inspiratory maneuver, such as PIF and TPF, exhibiting excellent specificity and sensitivity. Although DI showed a good AUC, it is practically nonfunctional in distinguishing SGS from nonstenosis considering that the cutoff value of 25 points is quite high, combined with a considerably lower sensitivity and specificity when compared to EDI. In our view, DI could further be used as a complementary measurement for assessing dyspnea in patients already diagnosed with upper airway obstruction, potentially affecting the decision-making and priority of a surgical intervention, as shown by other studies [17–19].

The major strengths of our study are the prospective inclusion of patients with different conditions and the standardized manner in which spirometry was performed in the whole study group. A potential limitation is the dissimilar size of the three cohorts, partly due to an inhibited inclusion rate during the COVID-19 pandemic. However, the data were sufficient to explore a clear pattern of spirometry findings in the respective groups. Finally, it could be presumed that, since the study population of SGS planned to undergo endoscopic intervention, it represented cases of relatively advanced disease suffering from severe dyspnea. However, repeating our analyses with only mild cases of SGS graded as Cotton-Myer 1 or even 2, we found no substantial difference compared to our primary results (data not shown). Subsequently, EDI should be part of the comprehensive work-up during the assessment of dyspnea of unclear cause that is unresponsive to treatment, since it can be useful to identify even mild severity of SGS.

## Conclusion

An increase in EDI in undiagnosed patients or nonresponders to empirical treatment for exertional breathing difficulties or dyspnea at rest could be a sign of upper airway obstruction and should be further assessed with laryngotracheoscopy.

### Supplementary Information

Below is the link to the electronic supplementary material.

Supplementary file1 (DOCX 15 KB)

Supplementary file2 (DOCX 18 KB)

## Data Availability

The datasets generated and/or analysed during the current study are available from the corresponding author on reasonable request.
