# Supplementary material for: How can we identify subglottic stenosis in patients with suspected obstructive disease?
Source: Eur Arch Otorhinolaryngol. 2023 Aug 4;280(11):4995–5001. doi: 10.1007/s00405-023-08141-3 (PMC10562276; doi:10.1007/s00405-023-08141-3)
Supplement: Supplementary file 1 — Supplementary file1 (DOCX 15 KB) [file 405_2023_8141_MOESM1_ESM.docx]

## Additional file legend:

Additional file 1: The original English version of the Dyspnea Index (with permission from Jackie Gartner-Schmidt).

## Additional file 1:

These are some symptoms that you may be feeling. Please circle the response that indicates how frequently you experience the same symptoms:

(0=never, 1=almost never, 2=sometimes, 3=almost always, 4=always)

| 1. | I have trouble getting air in |  | 0 | 1 | 2 | 3 | 4 |
| --- | --- | --- | --- | --- | --- | --- | --- |
| 2. | I feel tightness in my throat when I am having my breathing problem |  | 0 | 1 | 2 | 3 | 4 |
| 3. | It takes more effort to breathe than it used to |  | 0 | 1 | 2 | 3 | 4 |
| 4. | Changes in weather affect my breathing problem |  | 0 | 1 | 2 | 3 | 4 |
| 5. | My breathing gets worse with stress |  | 0 | 1 | 2 | 3 | 4 |
| 6. | I make sound/noise when I breath in |  | 0 | 1 | 2 | 3 | 4 |
| 7. | I have to strain to breathe |  | 0 | 1 | 2 | 3 | 4 |
| 8. | My shortness of breath gets worse with exercise or physical activity |  | 0 | 1 | 2 | 3 | 4 |
| 9. | My breathing problem makes me feel stressed |  | 0 | 1 | 2 | 3 | 4 |
| 10. | My breathing problem causes me to restrict my personal and social life |  | 0 | 1 | 2 | 3 | 4 |
