# Supplementary material for: How can we identify subglottic stenosis in patients with suspected obstructive disease?
Source: Eur Arch Otorhinolaryngol. 2023 Aug 4;280(11):4995–5001. doi: 10.1007/s00405-023-08141-3 (PMC10562276; doi:10.1007/s00405-023-08141-3)
Supplement: Supplementary file 2 — Supplementary file2 (DOCX 18 KB) [file 405_2023_8141_MOESM2_ESM.docx]

## Additional file 2 legend

Additional file 2: The Swedish version of the Dyspnea Index

## Additional file 2:

Här följer några symptom du kanske känner av. Sätt en ring runt den siffra som bäst motsvarar hur ofta du upplever de olika symptomen

(0=aldrig, 1=nästan aldrig, 2=ibland, 3=nästan alltid, 4=alltid)

| 1. | Jag har problem att andas in |  | 0 | 1 | 2 | 3 | 4 |
| --- | --- | --- | --- | --- | --- | --- | --- |
| 2. | Det känns trångt i halsen när jag har besvär med andningen |  | 0 | 1 | 2 | 3 | 4 |
| 3. | Det är mer ansträngande att andas nu jämfört med tidigare |  | 0 | 1 | 2 | 3 | 4 |
| 4. | När vädret ändras påverkas mina andningsbesvär |  | 0 | 1 | 2 | 3 | 4 |
| 5. | Jag har svårare att andas när jag blir stressad |  | 0 | 1 | 2 | 3 | 4 |
| 6. | Det låter när jag andas in |  | 0 | 1 | 2 | 3 | 4 |
| 7. | Jag måste anstränga mig för att andas |  | 0 | 1 | 2 | 3 | 4 |
| 8. | Mina andningsbesvär blir värre vid träning och fysisk aktivitet |  | 0 | 1 | 2 | 3 | 4 |
| 9. | Mina andningsbesvär gör att jag känner mig stressad |  | 0 | 1 | 2 | 3 | 4 |
| 10. | Mina andningsbesvär begränsar mitt personliga och sociala liv |  | 0 | 1 | 2 | 3 | 4 |
